# Leishmaniasis in the Colombian post-conflict era: a descriptive study from 2004 to 2019

**DOI:** 10.1590/0037-8682-0612-2020

**Published:** 2021-06-02

**Authors:** José Alejandro Iza Rodríguez, Shirley Natali Iza Rodríguez, Mario Javier Olivera

**Affiliations:** 1 Universidad Nacional de Colombia, Department of Medicine, Bogotá, Colombia.; 2 Universidad Militar Nueva Granada, Department of Medicine, Bogotá, Colombia.; 3 Instituto Nacional de Salud, Parasitology Team, Bogotá, Colombia.

**Keywords:** Leishmaniasis, Peace, Conflict, Epidemiology, Colombia

## Abstract

**INTRODUCTION:**

Leishmaniasis is strongly associated with armed conflict. We describe the epidemiology of leishmaniasis before and after the peace agreement in Colombia.

**METHODS:**

Data for 2004-2019 period were collected from the National Public Health Surveillance System. The annual incidence per geographical department before and after the peace agreement was calculated and correlated with armed conflict severity.

**RESULTS:**

The annual incidence of leishmaniasis registered a downfall with an annual percentage change of 17.7% after the peace treaty.

**CONCLUSIONS:**

A decrease in hostilities has a positive impact on the leishmaniasis incidence, which may be the case for other public health issues.

Leishmaniasis is a neglected disease caused by more than 20 *Leishmania* species. The disease is transmitted to humans by the bite of infected phlebotomine sand flies, which are predominantly from the genus *Phlebotomus* in the Old World and *Lutzomyia* in the New World^1^. Leishmaniasis is endemic in 97 countries distributed on every continent except Oceania and Antarctica, with focal areas of high prevalence in tropical and subtropical regions^2^.

In the Americas, cutaneous leishmaniasis (CL) is the most common clinical form and is endemic in 18 countries. In contrast, visceral leishmaniasis (VL), which is more severe and even fatal if left untreated, has a lower incidence. Mucocutaneous leishmaniasis (MCL) and diffuse cutaneous disease with chronic progression are less common than CL^3^ ^,^ ^4^. Both CL and MCL accounted for 989,096 new cases between 2001 and 2018 in the Americas^3^. The epidemiology of CL in the Americas depends on the regional ecological context and is highly sensitive to changes in the triad parasite-vector-reservoir^5^ ^,^ ^6^. CL has been depicted as an occupational disease, related to activities carried out in forests and other enzootic areas, and posing a high risk for military personnel when entering areas with high circulation of the vector insect^6^ ^-^ ^8^. 

For more than five decades (1964-2016), the Colombian armed conflict led to mass mobilization, from soldiers of the military forces, paramilitaries, and far-left guerrillas to civilians, all along the territory^7^ ^,^ ^9^ ^,^ ^10^. Before the “Final Agreement for the End of the Conflict and the Construction of a Stable and Long-lasting Peace” signed in 2016 between the National Government and the Revolutionary Armed Forces of Colombia-People’s (FARC-EP), fighters from all armed groups were highly affected by CL, with 50% of cases being soldiers of the National Army. Antileishmanial drugs restrictively controlled by the state, as a warfare strategy, showed a link between public health and armed conflict^9^ ^,^ ^10^. During the 1990 decade, approximately 6,500 leishmaniasis cases per year were reported, a number that increased progressively to reach 20,000 cases per year in 2005 and 2006, with CL accounting for the vast majority of cases (95-98%). Men (83% of cases) and those aged 15 to 44 years were mostly affected^7^ ^,^ ^11^. More than 45,000 cases of CL have been reported in soldiers between 2005 and 2010, and several CL outbreaks have affected army population^8^ ^,^ ^12^. From 2011 to 2017, CL cases in the army population dropped to 17,796^8^ ^,^ ^11^, the period in which the highest risk of infection was mainly reported in the departments of Antioquia, Norte de Santander, Santander, and Tolima^11^. According to the WHO, in 2017 and 2018, the incidence rate of CL decreased in the Americas^3^. In Colombia, new epidemiological dynamics may have resulted from post-agreement times. 

This descriptive study compared the data before and after the cease-fire agreement to determine the tendencies and features of leishmaniasis incidence in Colombia from 2004 to 2019, including the transitional period of peace in Colombia after the final agreement was signed in 2016. Data were obtained from morbidity records of the National Institutes of Health. Demographic information was obtained from population projections and series from the National Administrative Department of Statistics (DANE) webpage for the same period. Due to the availability of databases, information was obtained for the 2004-2019 period; although 2007 data were not available in the National Institute of Health when consulted, data taken for analysis resulted from a collection of data from the National Public Health Surveillance System (SIVIGILA) webpage.

For normally distributed data, values were reported as mean and standard deviation, while for non-normally distributed data, values were reported as median and interquartile range.

National incidence rates expressed as cases per 100,000 population per year were calculated based on data of cases, and for the denominator of the three clinical forms of leishmaniasis, the population at risk defined by the Ministry of Health and Social Protection of 11 million people was considered; for specific incidence by department, the projection of the rural population, according to the 2018 National Census, was considered as the denominator.

The absolute difference and percentage change in incidence between the previous and posterior peace agreement periods were calculated, as well as the mean change per year. Piecewise linear regression was used to identify whether there was a significant change in the trend slope. The change point was estimated using the maximum-likelihood approach.

The severity of the armed conflict by departments was quantified with the number of cases of war acts registered by department by the Observatory of Memory and Conflict from 1964 to 2016, and the strength of the association with CL cases was measured using the correlation coefficient.

All data were stored in a standard format in MS Excel (Microsoft, Redmond, USA) and analyzed using Stata (release 15, Stata Corporation, College Station, TX, USA). According to Colombian ethical normativity, this study was framed as research with no risk.

Over the 16-year study period, 166,609 new leishmaniasis cases were reported throughout the Colombian territory, with a mean annual incidence rate of 94 cases per 100,000 population, with a standard deviation of 34. CL represented 98% of the total cases, followed by MCL with 1% and VL with 0.3% (Figure 1).

**FIGURE 1:**
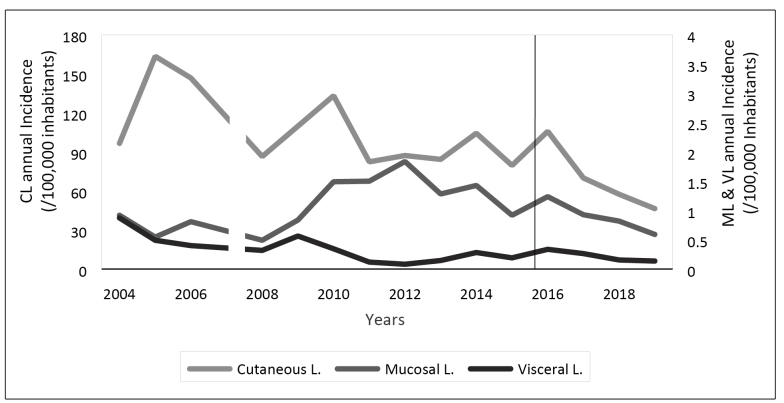
Time series analysis of annual incidence of Leishmaniasis between 2004 and 2019.

*CL: cutaneous leishmaniasis; **ML**: mucosal leishmaniasis; **VL**: visceral leishmaniasis.

In decreasing order, the departments with the largest leishmaniasis data during the study period were Antioquia, Meta, Tolima, Caquetá, and Santander, which together represented more than 52% of the national cases (during the 16-year period), which aligned with specific CL and MC geographical distribution. VL had a different spatial pattern, being specifically concentrated in the northern coastal region of Colombia, which corresponds to Bolivar, Cordoba, and Sucre, which together account for 80.7% of the total number of national VL cases. In these three departments, VL represented 2.6%, 3.3%, and 6.6% of the total leishmania cases, respectively, being significantly higher than the national figure (0.3%).

However, considering the population density per department, Guaviare, Vaupes, Caquetá, Vichada, and Putumayo presented the highest leishmaniasis incidence rates per 100,000 inhabitants during the study period. 

From 2004 to 2016, which represents part of the armed conflict period, the mean national incidence rate of leishmaniasis was 103 cases per 100,000 people per year, with a standard deviation of 32. Guaviare, Vaupes, Caquetá, and Meta had the highest incidence rates, which significantly surpassed the mean national rate. 

The years 2005, 2006, and 2010 presented incidence peaks, with national annual rates of 164 cases, 148 cases, and 135 cases per 100,000 inhabitants, respectively. Cases in Caquetá and Meta accounted for most of these peaks (Figure 2).

**FIGURE 2:**
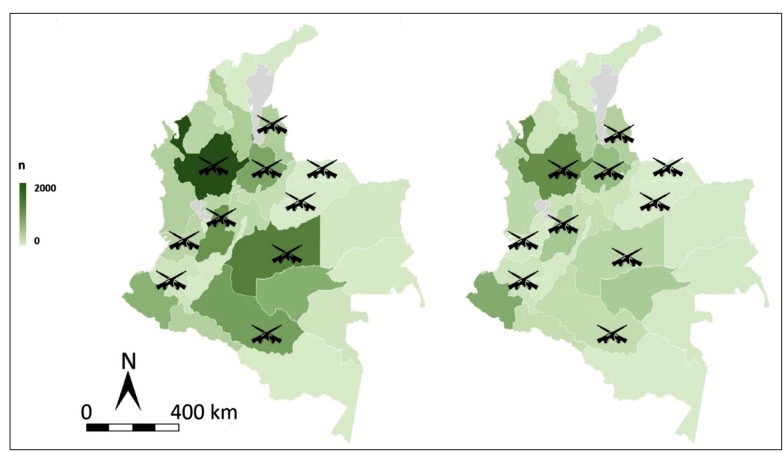
Geographical distribution before (right) and after (left) peace agreement.

*n: Annual incidence per 100,000 population at risk.

After the peace agreement was signed, although there was a significant decrease in total number of cases, the CL, MCL, and VL proportions were not different from those in the previous period. In decreasing order, the departments with the highest number of leishmaniasis cases in the last three studied years were Antioquia, Nariño, Santander, Tolima, and Guaviare.

The mean national annual incidence rate after the peace agreement dropped from 103 to 59 cases per 100,000 population, with a standard deviation of 12. This difference was 44 cases per 100,000 individuals (p = 0.039). The mean number of cases per year decreased by 4,841 cases between the armed conflict period and the post-agreement period (p = 0.039); the specific mean of CL cases per year decreased by 4,789 cases (p = 0.041).

By department, there was also a significant drop between the pre- and post-agreement periods; the mean number of cases of leishmaniasis decreased by 3,748 cases (p < 0.001), and CL cases presented a mean difference of 3,705 cases (p < 0.001).

The number of war acts by departments, reporting values of 651 (304-1528) for median and interquartile range, was higher in Antioquia, Cauca, Meta, Caquetá, and Santander departments. The strength of the association between armed conflict and CL cases was high (r = 0.766; p < 0.001).

Regression analysis showed a constant downward trend in the incidence of leishmaniasis. Between 2004 and 2016, there was a downward trend with an annual percentage change of 2.5%, which was not statistically significant (p = 0.324). Then it fell more intensely between 2016 and 2019, with an annual percentage change of 17.7%, which was statistically significant (p = 0.009).

Age, sex, and occupation data were not publicly available in the database. 

Although leishmaniasis persisted in the post-agreement period, the temporal analysis showed that the peace agreement significantly accelerated the rate of decrease in the incidence of the disease; a specifically positive impact was noted for CL, which may suggest a similar impact on other public health issues.

As the main official source of documentation about violence of the armed conflict in Colombia, the Observatory of Memory and Conflict registered geographically armed combat and war acts that best describe the influx of military personnel and guerrilla into the jungle scenarios, where they were exposed to the bite of the vector insect. Zones such as Antioquia, Meta, Caquetá, and Santander have been strategic areas, severely affected by guerrilla operations, and correspond with the highest CL rates before the peace agreement. The previously mentioned well-known geographical tendency corresponds to the high correlation found in this study. Regarding the density of cases per inhabitant, impoverished remote departments, such as Guaviare, Vaupes, Caquetá, Meta, and Vichada, presented the highest incidence rates. 

The peaks in CL cases in 2005, 2006, and 2010 could be a consequence of the Patriot Plan, the largest military offensive in the last decades developed by the Government of Colombia as a counter-guerrilla plan, which sought to obtain military presence in remote areas of Colombia with a 40% increase in troops in arms from 2002^13^. Along with this offensive of the public forces, the combat was forced to move to remote rural areas with high circulation of the vector insect.

Since the peace agreement, despite the cessation of armed combat against FARC-EP, the emergence of other criminal organizations considered remnants or successors of the dissolved guerrilla, along with persistent drug trafficking and poverty, has had some implications for the geographical configuration of the conflict, concentrating in certain areas, such as Antioquia, Nariño, and Santander, which is consistent with the recent distribution of CL.

The study period responds to data that are publicly available in databases. Although we analyzed MCL and VL, CL was mostly discussed due to a greater correlation with conflict. The major limitation of this study was information bias in the data and secondary sources. Another limitation was the lack of data from 2007 displayed in the National Institute of Health, which is exhibited in Figure 1; additionally, the information on the sociodemographic characteristics of the affected population such as occupation and vector information was not available.

The downward trend in leishmaniasis cases has been constant since the peace treaty. This trend will probably reach a plateau until vector control programs, along with prevention, detection, and treatment interventions surpassing transmission. Time will reveal new challenges in combatting leishmaniasis. More quantitative and qualitative studies are needed to understand the relationship between CL and residual conflicts after the 2016 peace agreement.

## References

[1] Claborn D (2010). The biology and control of leishmaniasis vectors. J Glob Infect Dis.

[2] World Health Organization (2019). Global Health Observatory data repository.

[3] Organización Panamericana de la Salud (2019). Leishmaniasis Informe Epidemiológico de las Américas.

[4] Arenas R, Torres-Guerrero E, Quintanilla-Cedillo MR, Ruiz-Esmenjaud J (2017). Leishmaniasis: A review. F1000Res.

[5] Pérez-Flórez M, Ocampo CB, Valderrama-Ardila C, Alexander N (2016). Spatial modeling of cutaneous leishmaniasis in the Andean region of Colombia. Mem Inst Oswaldo Cruz.

[6] World Health Organization (2010). Control of the leishmaniases. World Health Organ Tech Rep Ser.

[7] Instituto Nacional de Salud (2017). Consecuencias del Conflicto Armado en Salud en Colombia, Noveno Informe Técnico.

[8] Patino LH, Mendez C, Rodriguez O, Romero Y, Velandia D, Alvarado M (2017). Spatial distribution, Leishmania species and clinical traits of Cutaneous Leishmaniasis cases in the Colombian army. PLoS Negl Trop Dis.

[9] Pinto García L (2019). Leishmaniacs in lab coats and camouflage uniforms. Boletín informativo de la redLEISH.

[10] Pinto-García L (2019). Disentangling war and disease in post-conflict Colombia beyond technoscientific peacemaking. Tapuya Lat Am Sci Technol Soc.

[11] Instituto Nacional de Salud (2016). Informe del Evento Leishmaniasis.

[12] Vélez ID, Carrillo LM, López L, Rodríguez E, Robledo SM (2012). An epidemic outbreak of canine cutaneous leishmaniasis in colombia caused by Leishmania braziliensis and Leishmania panamensis. Am J Trop Med Hyg.

[13] Ávila AF (2008). FARC: dinámica reciente de la guerra. Arcanos Publicación de la Corporación Nuevo Arco Iris.

